# Professional nurses’ readiness for lifelong learning in Gauteng public healthcare: The motivation-barrier paradox

**DOI:** 10.4102/curationis.v49i1.2884

**Published:** 2026-09-30

**Authors:** Nirmala Chengalroyen, Charlene Downing

**Affiliations:** 1 Department of Nursing, Faculty of Health Sciences, University of Johannesburg, Johannesburg, South Africa

**Keywords:** barriers to learning, continuing professional development, lifelong learning, professional nurses, self-directed learning, public healthcare, South Africa

## Abstract

**Background:**

In South African public healthcare, continuing professional development (CPD) is essential for maintaining nursing competence; however, participation rates remain suboptimal despite regulatory mandates.

**Objectives:**

This study assessed professional nurses’ learning readiness and motivation, while reporting contextual factors influencing CPD participation in public hospitals in Gauteng province.

**Method:**

A cross-sectional design was employed with 158 professional nurses from three public hospitals, using validated instruments to measure self-directed learning readiness (17 items, *α* = 0.95), desire for learning (8 items, *α* = 0.92), and workplace learning opportunities. Descriptive statistics and exploratory factor analysis were conducted.

**Results:**

Despite 94.3% nurses having access to learning opportunities and 98.7% expressing interest, only 51.0% participated. Staff shortages emerged as the primary barrier (41.7%), followed by scheduling conflicts (16.7%). Nurses demonstrated strong learning readiness (mean 3.2–3.8/5.0), yet contextual constraints limited engagement. Technology deficits were pronounced: 84.8% lacked workplace internet access despite 82.5% preferring blended learning.

**Conclusion:**

The gap between high motivation and limited participation reveals that CPD challenges are fundamentally structural, not individual.

**Contribution:**

This study provides evidence that effective CPD in resource-constrained settings requires multilevel interventions addressing workforce adequacy, technology infrastructure, and organisational support.

## Introduction

Continuing professional development (CPD) represents a fundamental pillar of professional nursing practice, enabling nurses to maintain clinical competence, adapt to evolving healthcare demands, and deliver evidence-based patient care (World Health Organization [WHO] 2016). In South Africa, the South African Nursing Council (SANC) mandates CPD participation, requiring professional nurses to accumulate continuing education units throughout their careers (SANC 2018). However, despite regulatory requirements and the documented benefits of lifelong learning, CPD participation rates in public healthcare settings remain concerningly low (Bvumbwe & Mtshali 2018; Mlambo, Silén & McGrath 2021).

The fourth industrial revolution, global health challenges, including pandemic preparedness, and South Africa’s pursuit of Universal Health Coverage intensify the imperative for nurses to engage in continuous learning (National Department of Health [NDoH] 2020). Yet, professional nurses in public healthcare facilities face substantial barriers, including staff shortages, inadequate resources, and limited organisational support that constrain CPD engagement (Armstrong & Rispel 2015; Rispel & Bruce 2015). While international literature extensively documents facilitators and barriers to nurse CPD (Govranos & Newton 2014; Pool et al. 2016), context-specific empirical evidence from South African public healthcare remains limited.

Existing South African studies predominantly employ qualitative methodologies to explore perceptions and experiences (Bvumbwe & Mtshali 2018; Mlambo et al. 2021), without systematically measuring core constructs such as self-directed learning readiness and intrinsic learning motivation. This methodological gap constrains understanding of the relationship between individual learning dispositions and contextual factors, thereby limiting evidence-based intervention development. Furthermore, most studies focus either on individual nurse characteristics or organisational barriers, rarely integrating both perspectives to illuminate their dynamic interaction.

However, no South African quantitative studies have simultaneously measured nurses’ readiness for self-directed learning and empirically examined how organisational conditions constrain CPD participation. This knowledge gap limits the development of evidence-based interventions that address both individual capacity and systemic barriers.

This study addresses these gaps by systematically measuring professional nurses’ self-directed learning readiness and desire for learning, while concurrently exploring contextual factors influencing CPD participation in public hospitals in Gauteng province. Gauteng, South Africa’s most populous province and economic hub, hosts diverse public healthcare facilities serving predominantly resource-constrained communities, making it an ideal context for examining CPD dynamics in challenging settings.

### Research aim and objectives

The aim of this study was to measure professional nurses’ readiness for lifelong learning and identify contextual factors influencing CPD participation in Gauteng public healthcare settings. Specific objectives were to:

- Measure professional nurses’ self-directed learning readiness and desire for learning
- Examine the availability and accessibility of workplace learning opportunities
- Identify barriers preventing CPD participation
- Examine nurses’ learning preferences, technology access and future learning interests

### Significance of the study

This study contributes empirical evidence addressing critical knowledge gaps regarding CPD dynamics in resource-constrained public healthcare. By integrating psychometric measurement of learning readiness with contextual barrier analysis, findings inform multilevel interventions targeting individual capacity building, organisational support enhancement, and policy-level workforce planning. The results advance understanding of nursing management and workforce development, areas of critical importance in healthcare practice. The study’s significance extends beyond Gauteng to similar resource-limited settings globally where healthcare workforce development challenges persist.

## Literature review

### Conceptual framework: Lifelong learning in nursing

Lifelong learning in nursing encompasses formal continuing education, informal workplace learning, and self-directed professional development activities enabling nurses to maintain and enhance competence throughout their careers (Gopee 2005; Shahhosseini & Hamzehgardeshi 2015). Knowles’ andragogy theory provides a foundational understanding of adult learning principles, emphasising self-directed learning as central to professional development (Knowles, Holton & Swanson 2015). Self-directed learning readiness – the degree to which individuals possess attitudes, abilities, and personality characteristics necessary for autonomous learning – significantly influences CPD engagement (Fisher, King & Tague 2001; Guglielmino 1977).

This study employs an integrated conceptual framework to examine CPD engagement as the outcome of interactions between individual factors and contextual moderators. Individual factors comprise self-directed learning readiness and desire for learning (intrinsic motivation), representing nurses’ internal capacity and drive for professional development. Contextual moderators encompass organisational support dimensions (staffing adequacy, protected learning time, managerial encouragement), infrastructure availability (technology access, learning resources), and systemic conditions (workload pressures, financial constraints). The framework posits that high individual readiness is necessary but insufficient for sustained CPD participation; enabling contextual conditions are essential for translating motivation into action. This perspective aligns with situated learning theory (Lave & Wenger 1991), which emphasises that learning occurs within and is shaped by organisational and social contexts.

Intrinsic learning motivation, operationalised as desire for learning, represents the internal drive to engage in learning for inherent satisfaction rather than purely external rewards (Deci & Ryan 2000; Ryan & Deci 2020). Research demonstrates that nurses with higher self-directed learning readiness and intrinsic motivation engage more consistently in CPD activities and demonstrate superior clinical competence (Khodamoradi et al. 2019; Slater, Cusick & Louie 2017).

### Barriers to continuing professional development in resource-constrained settings

International literature identifies multiple barriers to nurse CPD, including time constraints, financial limitations, family responsibilities, inadequate organisational support, and accessibility challenges (Govranos & Newton 2014; Pool et al. 2016). In African contexts, these barriers intensify because of severe staff shortages, limited budgets, inadequate infrastructure and competing healthcare priorities (Bvumbwe & Mtshali 2018; Nsemo et al. 2013).

South African studies indicate that public sector nurses face particularly acute challenges. Armstrong and Rispel (2015) documented that nurse-patient ratios in Gauteng public hospitals frequently exceed safe levels, creating workload pressures that prevent learning participation. Rispel and Bruce (2015) identified inadequate managerial support and the absence of protected learning time as significant constraints. Mlambo et al. (2021) highlighted that while nurses value CPD conceptually, structural barriers render participation episodic rather than sustained.

Despite this evidence, limited research quantitatively measures the gap between nurses’ readiness for learning and actual participation rates, or systematically examines how organisational factors moderate the relationship between individual motivation and CPD engagement. This study addresses this gap through integrated measurement of individual dispositions and contextual constraints.

### Technology and contemporary continuing professional development

The coronavirus disease 2019 (COVID-19) pandemic accelerated the adoption of online and blended learning approaches in nursing CPD (Shen et al. 2021). While technology-enhanced learning offers flexibility, potentially overcoming traditional barriers, it requires adequate infrastructure and digital literacy (Rouleau et al. 2019). In resource-constrained settings, technology access inequities may create additional barriers, with CPD opportunities becoming accessible only to nurses with personal resources for devices and internet connectivity. Understanding nurses’ technology access and learning format preferences is essential for designing equitable contemporary CPD interventions.

## Research methods and design

### Study design and setting

A quantitative, descriptive, cross-sectional design was employed to measure professional nurses’ learning readiness and to explore contextual factors at a single time point. The study was conducted in three purposively selected Gauteng province public hospitals representing diverse contexts: Hospital A (tertiary, Charlotte Maxeke Cluster, 1000 beds), Hospital B (tertiary, Steve Biko Cluster, 800 beds), and Hospital C (district, George Mukhari Cluster, 200 beds). These hospitals serve predominantly resource-constrained communities and employ large professional nursing workforces.

### Population and sampling

The target population comprised all professional nurses employed in the selected hospitals (*N* = 270). Inclusion criteria were: (1) registered professional nurse with SANC, (2) employed full-time at study site, and (3) minimum 6 months’ experience at current facility. Exclusion criteria were: (1) student nurses, enrolled nurses, or nursing assistants, (2) nurses on extended leave and (3) temporary or locum staff.

Proportional stratified sampling ensured representation across hospital types and sizes. The target sample was 270 nurses (Hospital A: *n* = 135; Hospital B: *n* = 109; Hospital C: *n* = 26). The final sample comprised 158 respondents (Hospital A: *n* = 89; Hospital B: *n* = 53; Hospital C: *n* = 16), yielding a 58.5% response rate.

### Data collection instrument

A self-administered questionnaire comprised four sections:

**Section A: Demographic information:** Eight items capturing gender, age, ethnicity, work experience, hospital affiliation, and qualifications.

**Section B: Self-directed learning readiness:** Seventeen items adapted from Fisher et al.’s (2001) Self-Directed Learning Readiness Scale, rated on a five-point frequency scale (‘I feel this way all the time’ to ‘I feel this way less than usually’). Items measured learning autonomy, responsibility-taking, information-seeking skills, and theory-practice integration.

**Section C: Desire for learning:** Eight items adapted from Fisher et al.’s (2001) scale measuring intrinsic learning motivation, rated on a five-point agreement scale (‘Strongly agree’ to ‘Strongly disagree’). Items assessed learning enjoyment, persistence, openness to new ideas, and reflective practice.

**Section D: Workplace learning opportunities:** Mixed-format items (yes/no questions, multiple-choice, open-ended responses) explore availability of CPD opportunities, participation patterns, barriers encountered, learning preferences, technology access, and future learning interests.

Instrument adaptation involved language simplification for the South African context, removal of problematic items identified in pilot testing, and addition of context-specific barrier questions.

### Validity and reliability

Face and content validity were established through expert review by nursing education faculty (*n* = 3) and practising nurse managers (*n* = 2). Pilot testing with 30 professional nurses from non-study hospitals revealed initial reliability coefficients of *α* = 0.66 (Section B) and *α* = 0.71 (Section C). Item analysis identified poorly performing items that were revised or removed.

The main study’s reliability analysis demonstrated excellent internal consistency for Sections B (*α* = 0.95) and C (*α* = 0.92). Exploratory factor analysis of Section C revealed a single dominant factor explaining 65.9% of variance (Kaiser–Meyer–Olkin measure = 0.89; Bartlett’s test *χ*^2^(28) = 893.57, *p* < 0.001), confirming construct validity. All items demonstrated factor loadings > 0.62, indicating strong relationships with the underlying construct.

### Data collection procedure

Data were collected from June– to December 2025 using dual-mode administration (online via Google Forms and paper-based formats) to maximise accessibility. Nurse managers distributed study information and links to the questionnaire. Voluntary participation and anonymity were emphasised. Completed questionnaires were returned directly to researchers, bypassing management to ensure confidentiality.

### Data analysis

Data were analysed using Stata 17. Descriptive statistics (frequencies, percentages, means, standard deviations) summarised demographic characteristics and questionnaire responses. Reliability analysis employed Cronbach’s alpha coefficients and item-total correlations. Exploratory factor analysis used principal component factoring with Kaiser criterion (eigenvalue > 1) for factor retention. Exploratory factor analysis was prioritised for the desire for learning scale because of its adaptation and theoretical centrality to intrinsic motivation. Open-ended responses underwent content analysis with thematic categorisation and frequency counts.

### Ethical considerations

Ethical clearance to conduct this study was obtained from the University of Johannesburg’s Faculty of Health Sciences Research Ethics Committee on 14 November 2024. The ethical clearance number is REC-3223-2024; and Gauteng Department of Health (Protocol number: GP_202405_012). All participants provided written informed consent after receiving comprehensive study information. Participation was voluntary with no consequences for declining or withdrawing. Anonymity was maintained through coded responses without personal identifiers. Data were stored securely on password-protected devices accessible only to researchers. The study posed minimal risk to participants, involving only the completion of a questionnaire. No vulnerable populations were included. Findings will be disseminated through publications and presentations while maintaining participant confidentiality.

## Results

### Demographic characteristics

The sample (*n* = 158) comprised predominantly female (80.4%) and black African (94.9%) nurses of diverse ages (19.6% aged 21–29 years; 55.0% aged 30–44 years; 25.3% aged 45+ years). Work experience ranged from 1–5 years (15.2%) to 21+ years (13.9%), with the largest representation in the 6–10 years category (27.8%). Hospital distribution was: Hospital A 56.3% (*n* = 89), Hospital B 33.5% (*n* = 53), and Hospital C 10.1% (*n* = 16). The majority held nursing diplomas as a basic qualification, consistent with South Africa’s nursing education transition from diploma to degree pathways. Table 1 presents detailed demographic characteristics.

**TABLE 1:** Demographic characteristics of respondents (*n* = 158).

Characteristic	Category	Frequency (*n*)	Percentage
Gender	Female	127	80.4
	Male	22	13.9
	Prefer to self-describe	1	0.6
	Prefer not to say	8	5.1
Age group (years)	21–29	31	19.6
	30–44	87	55.0
	≥ 45	40	25.3
Ethnicity	Black African people	150	94.9
	White people	3	1.9
	Mixed race people	4	2.5
	Indian or Asian people	1	0.6
Work experience (years)	1–5	24	15.2
	6–10	44	27.8
	11–15	36	22.8
	16–20	32	20.3
	≥ 21	22	13.9
Hospital affiliation	Hospital A (Tertiary)	89	56.3
	Hospital B (Tertiary)	53	33.5
	Hospital C (District)	16	10.1

### Self-directed learning readiness

Section B responses revealed that nurses possess key self-directed learning attributes, although expression varies contextually. Across all 17 items, 67.7%–79.8% of responses selected ‘I sometimes feel this way’, with mean scores ranging from 3.2 to 3.8 on the five-point scale. Items indicating the strongest self-directed tendencies included:

- **Theory-practice integration (Item B9):** A total of 23.4% reported frequent ability to apply theoretical knowledge to practice.
- **Intrinsic** motivation **(Items B15, B16):** A total of 22.3% frequently want to learn new information; 22.2% frequently enjoy learning.
- **Responsibility-taking (Item B13):** A total of 20.3% frequently accept responsibility for making sense of learning.
- **Learning for its own** sake **(Item B8):** A total of 20.3% frequently love learning intrinsically.

Items indicating occasional dependency or challenges included:

- **Preference for external planning (Item B1):** Only 7.0% frequently prefer others to plan learning, suggesting nurses value autonomy.
- **Information location** difficulties **(Item B11):** Only 8.9% frequently experience difficulty locating information.
- **Reliance on external evaluation (Item B7):** Only 6.3% frequently believe others are better positioned to evaluate them.

The predominance of ‘sometimes’ responses suggests that while nurses possess self-directed learning dispositions, contextual factors influence whether these capacities manifest consistently in practice. Overall, the pattern reflects latent self-directed capacity constrained by contextual variability rather than absence of readiness.

### Desire for learning

Section C responses demonstrated strong endorsement of core professional learning values, although tempered by workplace realities. Across all eight items, 56.3%–63.9% selected ‘Neutral’, with combined positive responses (agree or strongly agree) ranging from 34.2% to 40.5%. Items with the strongest positive endorsement included:

- **Openness to new ideas (Item C22):** A total of 40.5% agreed or strongly agreed
- **Learning from mistakes (Item C19):** A total of 39.9% agreed or strongly agreed
- **Self**-evaluation **(Item C21):** A total of 38.0% agreed or strongly agreed
- **Evidence-seeking (Item C20):** A total of 37.3% agreed or strongly agreed

Disagreement was minimal across all items (0.6%–4.4%), indicating underlying positive learning orientations even among neutral respondents. The neutral response dominance suggests nurses conceptually value learning, but practical enthusiasm is tempered by contextual constraints.

### Workplace learning opportunities and participation

An overwhelming majority (94.3%, *n* = 149) reported that their workplace had provided learning opportunities post-qualification. However, among those with available opportunities, only 51.0% (*n* = 78) actually participated, while 47.7% (*n* = 73) did not participate despite availability. This reveals a critical *availability–accessibility gap*: opportunities exist, but substantial barriers prevent nurses from accessing them.

### Barriers to participation

Among the 73 nurses who did not participate despite available opportunities, content analysis revealed significant barriers, as presented in Table 2.

**TABLE 2:** Barriers to continuing professional development participation (*N* = 73).

Barrier category	Frequency (*n*)	%	Representative examples
Staff shortage	30	41.1	‘Staff shortage.’ (Hospital B, 30–34 years, Female) ‘Too little staff.’ (Hospital B, 21–24 years, Male) ‘I was the only RN on duty.’ (Hospital B, 40–44 years, Female) ‘The ward was short-staffed.’ (Hospital B, 21–24 years, Male)
Scheduling conflicts	12	16.4	‘was from night duty.’ (Hospital B, 30–34 years, Female) ‘I was day off on training day.’ (Hospital B, 40–44 years, Female) ‘I had just finished night shift.’ (Hospital A, 35–39 years, Female) ‘I had other work commitments.’ (Hospital B, 35–39 years, Female)
Selection or eligibility issues	10	13.7	‘I did not meet selection criteria.’ (Hospital C, 30–34 years, Female) ‘Only two nurses were selected.’ (Hospital B, 45–49 years, Female) ‘The Head Matron declined my application.’ (Hospital C, 30–34 years, Female) ‘I was told younger nurses have priority.’ (Hospital C, 45–49 years, Female)
Late notification or communication	9	12.3	‘I did not know about training until the day of training.’ (Hospital A, 35–39 years, Female) ‘I saw bulletin after closing date.’ (Hospital C, 21–24 years, Female) ‘I was not aware of training in time.’ (Hospital A, 30–34 years, Female)
Technology barriers	3	4.1	‘Internet access was down.’ (Hospital A, 35–39 years, Female) ‘It was online and I had no internet at home.’ (Hospital A, 30–34 years, Female) ‘There was no wifi for online course.’ (Hospital B, 50–54 years, Female)
Other	9	12.3	‘I had to self-fund.’ (Hospital B, 30–34 years, Male) ‘Training cancelled at short notice.’ (Hospital B, 45–49 years, Female) ‘Patient load too much.’ (Hospital B, 30–34 years, Female)

**Total**	**73**	**100.0**	**-**

Staff shortage emerged as the predominant constraint (41.1%), preventing nurses from attending even when motivated and when opportunities existed. This finding is critical: *the primary barrier is structural/systemic rather than individual*.

### The motivation-barrier paradox

Figure 1 illustrates the dramatic gap between availability of opportunities, interest in learning and actual participation – demonstrating the motivation-barrier paradox central to this study’s findings.

**FIGURE 1:**
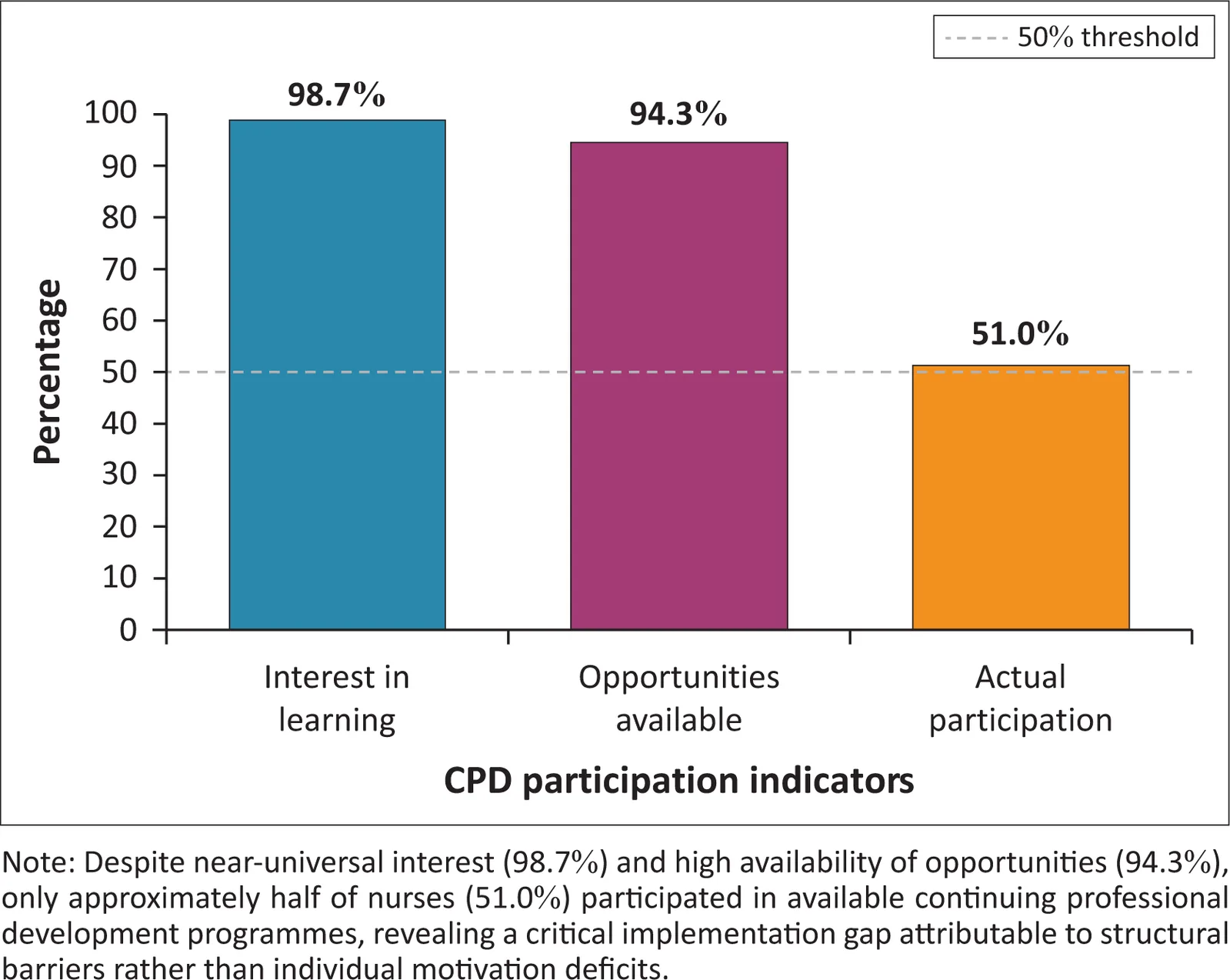
The motivation-barrier gap in continuing professional development participation (*N* = 158).

This visualisation demonstrates that the problem is not a lack of motivation (98.7% interested) or a lack of organisational provision (94.3% have opportunities), but rather systemic barriers that prevent nurses from accessing available opportunities.

### Learning interests and preferences

Future learning interest was nearly universal (98.7%, *n* = 153), with only two nurses expressing disinterest – both because of systemic barriers (selection inequity and approaching retirement) rather than a genuine lack of interest. This confirms exceptionally high motivation despite participation barriers.

Learning frequency preferences strongly favoured monthly opportunities (90.4%) over weekly (9.6%), likely reflecting workload realities. Format preferences were predominantly blended learning combining face-to-face and online (82.5%), with only 14.3% preferring face-to-face exclusively and 2.6% online exclusively.

Topics of interest revealed extraordinary diversity (over 100 distinct topics mentioned), with highest frequencies for:

- Critical care nursing (7.8%)
- Trauma nursing (5.9%)
- Paediatric and neonatal care (6.5%)
- Midwifery and maternal care (6.5%)
- Primary healthcare (6.5%)
- Public health (5.2%)
- Quality control and management (5.2%)

This diversity indicates that CPD models must accommodate varied interests across clinical specialties, management, public health, and emerging technologies rather than standardised programming.

### Technology infrastructure

Despite 82.5% preferring blended learning formats, significant technology infrastructure deficits were evident, as presented in Table 3.

**TABLE 3:** Technology infrastructure access for online learning (*N* = 158).

Technology resource	Available		Not available	
	*n*	%	*n*	%
Workplace internet access	24	15.2	134	84.8
Workplace computer/laptop access	25	15.8	133	84.2
Nurses who prefer a blended learning format	130	82.5	28	17.5

This technology gap creates a critical mismatch between preferred learning formats (82.5% want blended learning) and available infrastructure (84% lack workplace internet/computers), potentially shifting costs to individual nurses and creating socioeconomic inequities.

### External learning participation

A substantial 62.2% (*n* = 97) had participated in learning opportunities outside the workplace, indicating nurses actively seek CPD beyond employer-provided programmes. This demonstrates not only strong intrinsic motivation but also highlights potential inequities favouring nurses with financial and temporal resources to pursue external learning.

## Discussion

This study reveals a profound paradox: professional nurses in Gauteng public hospitals demonstrate exceptionally high motivation for lifelong learning (98.7% express interest) and possess strong self-directed learning readiness (mean scores 3.2–3.8/5.0) and positive learning orientations (minimal disagreement with learning values), yet only half (51.0%) participate in available CPD opportunities. This motivation-barrier gap, visualised in Figure 1, constitutes the study’s most significant finding, demonstrating that limited CPD participation reflects *structural constraints rather than individual deficits*.

### The primacy of systemic barriers

Staff shortage emerged as the dominant barrier (41.1% as shown in Table 2), far exceeding other constraints. This finding aligns with documented nurse–patient ratio challenges in South African public healthcare (Armstrong & Rispel 2015) but empirically quantifies its specific impact on CPD participation. The prevalence of this barrier reveals that adequate staffing is not merely a workforce management issue but a *foundational prerequisite for professional development*. When releasing nurses for training compromises patient care, organisations face impossible choices between immediate service delivery and long-term workforce competence.

This finding has profound implications for CPD policy. Traditional approaches emphasising training programme development or individual motivation enhancement are insufficient when the healthcare system’s fundamental staffing crisis prevents participation. Effective CPD facilitation requires integrated workforce planning addressing both adequate staffing levels and protected learning time, challenging the siloed approach in which staffing and professional development are managed separately.

### Context-dependent readiness

The predominance of ‘sometimes’ responses (67.7%–79.8%) across self-directed learning items reveals that readiness is not a stable trait but a *context-dependent capacity*. Nurses possess self-directed learning dispositions – evidenced by strong endorsement of intrinsic motivation (22.2%–23.4% frequent), responsibility-taking (20.3%) and theory-practice integration (23.4%) – but express these behaviours variably depending on organisational conditions. This pattern aligns with situational learning theory (Lave & Wenger 1991), suggesting self-directed learning emerges from interactions between individual capacity and environmental affordances.

This finding challenges individualistic CPD interventions focused solely on enhancing nurse readiness. While nurses demonstrate foundational readiness, translating this into consistent learning behaviour requires organisational enablers: manageable workloads, accessible resources, protected time and supportive cultures. The concept of *readiness* must be reconceptualised not as an individual attribute to be developed but as a *contextually enacted capacity* requiring supportive conditions.

### The technology infrastructure deficit

The gap between learning format preferences (82.5% want blended learning, as shown in Table 3) and infrastructure availability (84% lack workplace internet and/or computers) represents a critical contemporary challenge. As CPD increasingly incorporates online components (Rouleau et al. 2019; Shen et al. 2021), technology infrastructure becomes foundational rather than supplementary. The current deficit creates inequitable access where online learning depends on personal resources – devices, home internet, data costs – thereby disadvantaging nurses with limited financial means.

This finding extends beyond infrastructure provision to raise fundamental equity concerns. When contemporary CPD assumes access to technology, structural inequities in South African society (income disparities, geographic digital divides, differential home connectivity) directly translate into professional development inequities. Organisations must either provide workplace technology infrastructure enabling equitable access or risk entrenching socioeconomic disadvantages into professional advancement opportunities.

### Organisational support as multidimensional

Findings reveal that organisational support extends beyond opportunity provision to encompass multiple enabling dimensions, as evidenced in Table 2:

- *Adequate staffing* for releasing nurses without compromising care (41.1% cited staff shortage)
- *Scheduling systems* accommodating shift work (16.4% cited scheduling barriers)
- *Equitable selection processes* preventing arbitrary exclusions (13.7% faced selection barriers)
- *Effective communication* ensuring timely notification (12.3% cited late notification)
- *Technology infrastructure* enabling contemporary learning formats (Table 3)
- *Protected learning time* embedded in workforce planning

Current organisational support is *incomplete:* opportunities exist (94.3%), but enabling conditions often do not. This incompleteness explains the participation gap illustrated in Figure 1. Comprehensive organisational support requires simultaneous attention to all dimensions rather than piecemeal interventions.

### External learning as compensation strategy

The finding that 62.2% pursue external learning demonstrates nurses’ commitment to professional growth despite workplace barriers. However, this self-directed compensation strategy raises equity concerns. Nurses with financial resources, supportive families, and flexible personal circumstances can supplement inadequate workplace CPD through external courses, conferences or online programmes. Those lacking these resources experience compounded disadvantage: workplace barriers prevent access to organisational CPD, while personal constraints prevent access to external alternatives. This dynamic may contribute to competency gaps that correlate with socioeconomic factors rather than professional commitment.

### Implications for continuing professional development model development

These findings necessitate reconceptualising CPD facilitation from individual-focused interventions towards *multi-level ecological models* addressing four interconnected levels:

- **Individual level:** Maintaining and nurturing intrinsic motivation and self-directed learning dispositions that already exist among nurses.
- **Organisational level:** Creating comprehensive enabling conditions, including adequate staffing, protected time, equitable selection, effective communication, and technology infrastructure.
- **Policy level:** Integrating professional development planning with workforce planning, mandating staffing levels that enable learning participation, and funding technology infrastructure as an essential CPD enabler.
- **System level:** Addressing fundamental healthcare system challenges (chronic underfunding, staff shortages and resource constraints) that undermine all professional development efforts.

Effective CPD facilitation requires simultaneous intervention across all levels. Individual motivation enhancement without organisational enablement merely widens the motivation–participation gap, while organisational programming without addressing systemic workforce challenges creates opportunities nurses cannot access.

### Study’s strengths and limitations

Study strengths include validated instruments with excellent reliability (*α* = 0.92–0.95), a diverse sample across hospital types and career stages (Table 1) and integration of psychometric measurement with contextual barrier analysis. The 58.5% response rate, while lower than anticipated, provided adequate statistical power for descriptive and exploratory analyses.

Limitations include a cross-sectional design precluding causal inference, reliance on self-reported data susceptible to social desirability bias (although frequency framing may have mitigated this), ethnic homogeneity (94.9% black African, as shown in Table 1) constraining diversity analysis and a Gauteng-specific context potentially limiting transferability. In addition, the study measured readiness and motivation but not actual learning outcomes or competency improvements, leaving the relationship between CPD participation and professional competence unexamined.

## Conclusion

This study empirically demonstrates that professional nurses in Gauteng public healthcare possess strong readiness for lifelong learning and exceptional motivation for professional growth. The critical challenge is not nurse capacity or commitment but rather *systemic barriers that prevent nurses from accessing available CPD opportunities*. Staff shortage, as the predominant barrier affecting 41.1% of non-participants (Table 2), reveals that effective CPD facilitation requires fundamental healthcare system strengthening rather than merely enhanced training programmes.

The motivation-barrier paradox documented in this study (Figure 1) – 98.7% interested, 94.3% have opportunities, yet only 51.0% participate – challenges simplistic assumptions that limited CPD engagement reflects nurse disinterest or inadequate organisational provision. Instead, findings reveal complex implementation challenges requiring multilevel interventions addressing individual readiness maintenance, comprehensive organisational support, adequate workforce planning, technology infrastructure investment (Table 3) and fundamental healthcare system strengthening.

As South Africa pursues Universal Health Coverage and responds to evolving healthcare demands, understanding these dynamics becomes essential for developing evidence-based CPD strategies that genuinely support professional nurse development while acknowledging the structural realities of public sector practice. Future research should examine longitudinal relationships between organisational support dimensions and sustained CPD participation, explore interventions addressing identified barriers and investigate whether enhanced CPD engagement translates into improved patient outcomes.

This study provides foundational evidence for reconceptualising CPD facilitation in resource-constrained contexts, shifting focus from individual deficits towards creating systemic conditions that enable nurses to translate their motivation and readiness into sustained professional development.
